# Purification of ultrasonic assisted extracted chlorogenic acid from *Citrullus lanatus* rind using macroporous adsorption resin (MPAR)

**DOI:** 10.1016/j.fochx.2025.102374

**Published:** 2025-03-19

**Authors:** Sobia Naseem, Muhammad Rizwan, Wahidah H. Al-Qahtani, Ayesha Sadiqa, Awais Ahmad

**Affiliations:** a Department of Polymer & Process Engineering, University of Engineering and Technology Lahore, Pakistan; b Department of Chemistry, University of Engineering and Technology Lahore, Pakistan; c Department of Food Sciences & Nutrition, College of Food & Agriculture Sciences, King Saud University, Riyadh 11451, Saudi Arabia; d Department of Chemistry, University of Lahore, P.O. Box 54000, Lahore, Pakistan

**Keywords:** Chlorogenic acid, Watermelon (*Citrullus lanatus*) rind, Macroporous adsorption resin, Food waste

## Abstract

Recycling food waste to extract valuable bioactive compounds (BAC) offers a groundbreaking solution to mitigate environmental challenges. The isolation and purification of BAC from food waste are getting more attention to maintain environmental sustainability. The therapeutic potential of these compounds highlights the need for innovative approaches to extracting and purifying them from by-products. In this context, the chlorogenic (CG) acid is extracted from watermelon *(Citrullus lanatus)* rind (WMR) using ultrasound-assisted extraction (USAE) technique and purified using the macroporous adsorption resin (MPAR) method. The extraction and purification were carried out with water, 70 % *v*/v ethanol/water (70 % Et), and 90 % v/v ethanol/water (90 %-Et) solvent to analyze the impact of solvent on extraction efficiency, purity, and recovery. The extracted CG acid was qualitatively analyzed via UV spectrometry and HPLC and underwent FESEM analysis while purified CG acid was characterized via FTIR and thermal analysis. The implementation of the MPAR method notably increased the yield to 4.6 mg/g of raw material using solvents comprising 90 % ethanol than water and 70 % ethanol. The recovery of CG acid significantly escalated to 88 %, enhancing the purity percentage from an initial range of 70–94 %.

## Highlights

• Chlorogenic (CG) acid was extracted using an ultrasound-assisted extraction technique.
• CG acid was subsequently purified using macroporous adsorption resin (MPAR) method.
• Extraction & purification were carried out using water, 70 %-Et,and 90 %-Et solvent.
• Implementation of MPAR method has increased the yield, recovery and purity of CG acid.
• Recovery of CG acid escalated to 88 %, and purity from 70 to 94 % using 90 %-Et solvent.

## Graphical abstract

**Figure:**
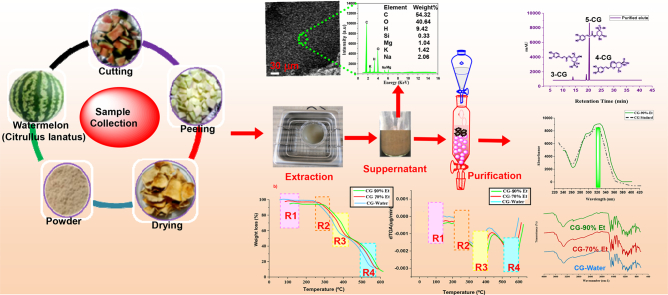

## 1 Introduction

The extraction of bioactive compounds from food waste is an emerging strategy to achieve zero waste while addressing critical environmental and economic challenges. Food waste, often overlooked, is a valuable source of bioactive compounds such as dietary fibers, polyphenols, flavonoids, and carotenoids, which possess significant health benefits. Traditional Chlorogenic (CG) acid extraction has relied on food sources such as coffee beans and sunflower seeds, which raises concerns regarding food security and resource allocation. Extracting CG acid from fruit waste such as watermelon rind presents an eco-friendly and cost-effective alternative. This not only mitigates waste accumulation but also capitalizes on underutilized agricultural byproducts to obtain high-value compounds. Exploring advanced and sustainable extraction techniques not only minimizes the environmental footprint associated with waste disposal but also opens new avenues for the development of pharmaceuticals, functional foods, and nutraceuticals. This approach aligns with the principles of the circular economy, promoting resource efficiency, sustainability, and waste valorization by converting discarded materials into high-value products (Zhao et al., 2021).

Chlorogenic (CG) acids are a group of phenolic compounds, derived from the esterification of quinic acid with any one compound of the trans-cinnamic acids, including ferulic, coumaric, and caffeic acids, hence it is also named Caffeoylquinic (CQ) acid (Lu et al., 2020)(Article & Pradesh, 2019). It is a secondary metabolite found in a variety of foods, such as fruits, vegetables, and herbal medicines. These include honeysuckle, potatoes, cork, Eucommia leaves, chrysanthemum, strawberries, mangoes, blueberries, leaves of mulberry, and green coffee beans (Lu et al., 2020).

Watermelon (*Citrullus lanatus*) is a popular fruit crop that is a great source of minerals, flavonoids, amino acids, vitamins, and phenolic compounds (especially chlorogenic acid and sinapic acid) (Raliya et al., 2016), which have strong anti-peroxyl radical properties. Watermelon rind, an abundant agricultural byproduct, is a rich source of bioactive compounds, including chlorogenic acid, which exhibits antioxidant, anti-inflammatory, and metabolic health benefits. Genetics and environmental variables such as harvest ripeness, post-harvest management, and agricultural techniques affect the phenolic content in pulp and rind (Ndikau et al., 2017).

Extraction of phenolic compounds using green, sustainable advanced techniques like ultrasound and microwave-assisted extraction, has been a common practice for a couple of decades (Xie, Zhao, et al., 2023). Moreover, their purification from crude extract to get high yield purity is the course of attention. The purification has been conducted through either aqueous two-phase, membrane filtration, centrifugation partition chromatography, etc., or column chromatography using ionic liquid (W. Wang et al., 2021). These methods are limited in their implications owing to high cost, low efficiency, and chemical overconsumption. To mitigate these concerns, the macroporous adsorption resin (MPAR) (Ratnaningsih et al., 2021) method is the focusing part of researchers due to its highly selective, productive, high adsorption capacity, and precise method that is operated at mild conditions (Ren et al., 2025; Solarte-Toro et al., 2018). Previous studies have successfully employed MPAR for phenolic compound purification, but its application for chlorogenic acid extraction from *C. lanatus* rind remains unexplored. This study aims to optimize MPAR-based purification, exploring its advantages to develop an efficient and sustainable approach for chlorogenic acid recovery.

This study aimed at the extraction of CG acid from watermelon rind (WMR) using an ultrasound-assisted extraction technique and its purification through MPARS to get maximum yield and purity. The extraction and purification were carried out with water, 50 %, and 70 % ethanol solvent to analyze the impact of solvent on extraction efficiency, purity, and recovery of CG acid. Among polar and non-polar MPARs, the NKA-II is selected for the purification of CG acid crude extract because adsorbent and adsorbate have a polar nature and must have a strong bonding during adsorption. This is the pioneering research to extract chlorogenic acid from WMR using the USAE technique and subsequently purify it to level up the yield, recovery, and purity percentage using MPAR; the safe and reliable method to secure the economic value. The purified CG acid could potentially be used for the medicinal or part of the food industry owing to its therapeutic role.

## 2 Procedure

### 2.1 Material and method

**Watermelon** (*Citrullus lanatus*) obtained from a supermarket in Pakistan. Ethanol, acetonitrile, and hydrochloric acid of lab grade were purchased from Sigma Aldrich Germany. The standard chlorogenic acid (3-CG acid, 4-CG acid, and 5-CG acid) and gallic acid (GA) of HPLC grade were purchased from Aladdin Bio-Chem Technology, China. NKA-II as a (MPAR) purchased from Cangzhou, Co. Ltd. China.

### 2.2 Isolation and drying of watermelon rind (WMR)

The watermelon's rinds were collected after the complete removal of the edible portion. These rinds were washed two times using distilled water to get rid of any dust and contaminations. Subsequently, the rind is cut into small pieces measuring approximately 2 cm using a knife and spread cleaning towel to remove excess water (Xie, Chong, et al., 2023). Afterward, they were spread on trays of the dryer and subjected to mechanical drying using a Cabinet dryer (OV-165-dryer, Gallen Kamp) operated at 100 °C and 0.6 m/s air velocity for 6 h (Raliya et al., 2016). After collecting dried watermelon rinds (WMRs) having moisture content up to 4 %, they were ground to a fine powder for further processing of extraction.

### 2.3 CG acid extraction

**Fig. 1:**
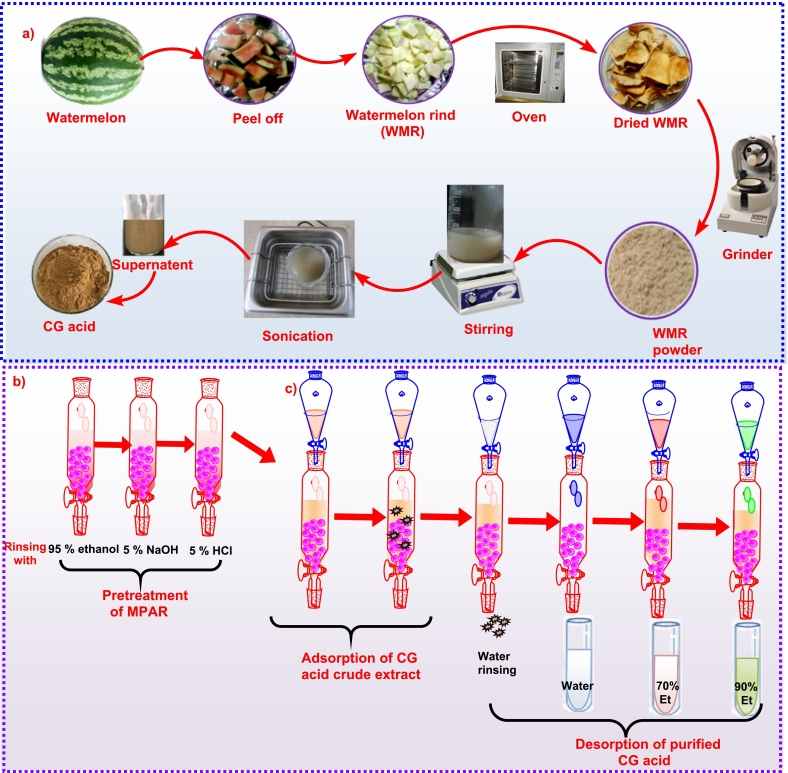
a) Extraction of chlorogenic (CG) acid from watermelon *(Citrullus lanatus)* rind, b) preparation of MPAR (NKA-II), c) Purification of crude CG acid extract using MPAR.

The CG acid was extracted from dried watermelon rind (WMR) powder with some modifications (Alves Filho et al., 2020). 10 g of WMR powder dissolved into water, 70 % and 90 % aqueous ethanolic solution under ultrasonic waves using an ultrasonic bath (5 L-230 V-25 KHz Bio-Techno Lab, Mumbai). The extraction was conducted at 10:1, 15:1, and 20:1 mL/g solvent-to-sample ratios to optimize the extraction yield of CG acid (Xie, Chong, et al., 2023)(Zeng et al., 2022). The mixture was sonicated at 150 W powder with constant temperature conditions set at 70 °C. and three extraction cycles of 30 min each. The process operates at an ultrasonic frequency of 20 kHz with a power density of 30 W/L (Chadni et al., 2022). The phosphoric acid is used to adjust the pH as a buffering agent. After the extraction, the extract was allowed to cool down to room temperature and followed the centrifugation at 2000 rpm for 15 min. The supernatant was collected into a sterilized bottle and stored at a temperature of 4 °C until further use in subsequent experiments or analysis. Fig. 1a shows the complete procedure of extraction of CG acid from the watermelon (*Citrullus lanatus*) rind.

### 2.4 Phenolic compounds analysis

The total phenolic content (TPC) of all CG acid extract samples before and after purification was determined by following the method with some modifications described in (Y. Chen et al., 2022) using gallic acid equivalent (GAE). The 50 mL of sample extracts were mixed with 400 mL of Folin Ciocalteu reagent and 10 mL of water and the solution was kept for 10 min. The solution was topped with 700 mL of sodium carbonate and incubated for 1 h at room temperature and underwent UV analysis using a spectrophotometer (U-5100, Tokyo, Japan) at 730 nm wavelength. The TPC of CG acid samples was determined in mg of GAE/g of CG acid extract on a dry weight base (Náthia-Neves & Alonso, 2021a).

### 2.5 Quantification of CG acid using HPLC

For determining the CG acid quantity, 1 mL of supernatant of crude extract after sonication and eluent obtained after purification were mixed with 10 mL of deionized water to dilute it and stirred electromagnetically for 10 min. This diluted solution subsequently passed through the column of HPLC at a pre-adjusted 329 nm wavelength (Zeng et al., 2022). Similarly, the standard solutions of CG acid at different concentrations (2 mg 4 mg, 6 mg, 8 mg, 10 mg, 12 mg, 14 mg, and 16 mg) were prepared and subjected to HPLC analysis to get a calibration curve. The graph has a linear relation of standard CG acid concentration (on the X-axis) and absorbance (on the Y-axis) with regression constant (R^2^ = 0.9999) value. The CG acid content of the extracted supernatant was determined by comparing its value on calibration and calculating the concentration of unknown CG acid solution by placing the value of its absorbance (Y-valueX in Y = 0.0044 x + 0.0488 equation. The results are expressed in milligrams (mg) of chlorogenic acid per gram of the sample. Moreover, the extraction of CG acid was confirmed through the comparative study of crude extracted and purified eluent with a spectrum of standard CG acid (X. Wang et al., 2019).

### 2.6 Extraction efficiency

The extraction efficiency of each crude extract (CG-water, CG-70 %-Et, CG-90 % Et) and eluent after purification (CG-water-p, CG-70 %-Et-p, CG-90 % Et-p) can be determined in terms of yield extraction, purity (%), and recovery of CG acid. Eq. 1 is used to find out the yield of CG acid (Bouhzam et al., 2023).

(1) $$\mathrm{Yield}\left(\mathrm{mg}/g\right)=\frac{\mathrm{Conc}.\mathrm{ofCGacid}\left(\mathrm{mg}/\mathrm{mL}\right)\times \mathrm{Vol}.\mathrm{ofExtract}\;\left(\mathrm{mL}\right)}{\mathrm{Weightofrawmaterial}\;\left(g\right)}$$

(2) $$\mathrm{Purity}\;\left(\%\right)=\frac{\mathrm{WeightofCGacidin}\;\mathrm{dry}\;\mathrm{extract}\left(\mathrm{mg}\right)}{\mathrm{Weight}\left(g\right)\mathrm{of}\;\mathrm{dry}\;\mathrm{extract}}\times 100$$

(3) $$\%Re\mathrm{covery}=\frac{\mathrm{CGacidcotentinextract}}{\mathrm{Weightofsample}}\times 100$$

### 2.7 HPLC analysis of CG acid

The crude extract and eluent of purified CG acid of each sample (CG-water, CG-70 %-Et, and CG-90 % Et) was analyzed through HPLC –DAD (Shimadzu LC-10 A, Germany) following the protocol described by the (Náthia-Neves & Alonso, 2021a). The 1 g of CG acid powder was mixed with 10 mL of 10 % of methanol. Moreover, the quantity of antioxidant preservative (butyl hydroxy-toluene) was added to a minimal fraction around 0.4 g to avoid oxidation of CG acid (Mushtaq et al., 2015). The extracts were injected in the HPLC column (Merck, Darmstadt Germany) c-18 (4.6 mm × 150 mm) and connected with a diode array detector (DAD) (Rivera-Mondragón et al., 2019). Before injection, the sample extracts underwent centrifugation at 4000 rpm, sonication for 5 min, and filtration using a syringe filter with a diameter of 0.45 μm for its smooth running, and avoiding any coagulation and trapping of air bubbles Shi et al., 2022. The gradient of the solvent phase was developed by mixing solvent 1 (acetic acid and water in a 6:94 ratio) with 100 % acetonitrile (solvent 2) and fluxed at 1.1 mL/min. The CG acid was eluted by using a gradient of solvent 1 starting with treating 10 %, starting, 40 % after 5 min till 6 min, and 100 % solvent used till 7–8 min. The study was conducted at 30 °C and the detection wavelength adjusted at 329 nm (Kim et al., 2020)

### 2.8 LC-ESI/MS analysis of purified CG acid

LC-ESI/MS analysis of purified CG acid sample collected from 200 to 400 mL desorption eluent volume was analyzed using liquid chromatography (Shimadzu 2010 LCMS) coupled with a mass spectrometer (Triple TOF 5600, AB SCIEX, Singapore). The autosampler was attached with two pumps, for running of solvent 1 (0.4 % aqueous solution of formic acid) and solvent 2 (methanol) at 500 μL/min flow rate. To detect peaks, a DAD detector scanned the chromatogram at 200–600 nm while recording at 255 nm and 329 nm wavelengths (Ayouaz et al., 2021). Electron spray ionization (ESI) was used as the ion source for the mass spectrometer. The mobile phase gradient increases linearly over 45 min from 10 % B to 70 % B, stays at that level for the following 10 min, and then reverts to 10 % B by the 75 min for rebalancing. Thereafter, there is a 10-min isocratic interval for re-equilibration.

### 2.9 Macro porous adsorption resin (MPAR) purification

#### 2.9.1 Pretreatment of MPAR

The CG crude extract was purified and isolated by using macroporous adsorption resin (MPAR) mainly NKA-II as the literature supported its efficient results for adsorption desorption capacity. NKA-II is composed of a cross-linked styrene-divinylbenzene copolymer. It is designed with suitable pore size and specific surface area, providing good selectivity for saponins and flavonoids. Before its use, it needs some pretreatment to maintain its moisture content. The NKA-II resin was dipped in 95 % ethanol solution for 24 h and subsequently washed with 5 % aqueous solution of sodium hydroxide, 5 % hydrochloric acid, and double distilled water alternatively. Following this pretreatment, a thorough washing of resin with distilled water is also required to get turbid-free resin (Bai et al., 2019). The pretreatment of MPAR is shown in Fig. 1b. The physical properties of NKA-II are listed in Table 1S.

#### 2.9.2 Adsorption and desorption test

The dynamic adsorption and desorption test was conducted according to the method of Fu et.al, (Fu et al., 2021) with some modifications. The crude extract of CG acid was purified by using NKA-II as macroporous adsorption resin (MPAR) in the resin column chromatography (RCC) technique. 3 g of resin filled with wet packaging has 50 BV bed volumes in glass column dimensions (400 mm × 18 mm × 8.8). The crude extract of CG acid (5 g/50 mL) passed through the column with a 1.5 BV/h flow rate. The resin column was kept at 25 °C for 5 h to get adsorption equilibrium. After attaining the equilibrium point of adsorption, the column was flushed with distilled water (DW) using 1 mL of bed volume to get impurity-free adsorbed CG in the resin column. The complete procedure of purification through adsorption and desorption on MPAR (NKA-II) is shown in Fig. 1c.

The CG acid of three samples was eluted from the column using 4 mL of bed volume for each of the water, 70 % ethanol, and 90 % ethanol solvents and labeled them CG-water, CG-70 %-Et, and CG-90 % Et. The eluent of each sample was collected and underwent UV spectrometry and HPLC analysis. The adsorption-desorption cycle (ADC) experiment was conducted thrice. The eluent was concentrated and frozen under N_2_ gas at −20 °C and then dried using a freeze drier (Christ Alpha 1–2 LD plus Osterode, Germany) to calculate yield, purity (%), and percent recovery.

#### 2.9.3 Adsorption kinetics and isotherm

The three samples of crude extract (20 mL) of CG acid were poured into the flask containing 2 g of NKA-II resin (Meng et al., 2023). The temperature of the mixture was regulated by placing it in a shaker with 150 rpm speed for 6 h. The quantification of CG acid crude extract was analyzed after 20 min up to 6 h. The kinetic studies were designed to analyze the adsorption rate of the sample on macroporous adsorption resin (MPAR) (Hou & Zhang, 2021). Two kinetic models were studied to elucidate the fitting of experimental data. The 1st order and 2nd order kinetics are given below in Eqs. (4), (5) respectively.

(4) $$\ln\left(q_{e}-q_{t}\right)=-k_{1}t+\ln q_{e}$$

(5) tqt=1k2q2+tqe

where q_e_ and q_t_ are the adsorption capacity (mg/g) at equilibrium and time t and k_1_ (1/min) and k_2_ (g/mg.min) are rate constant for pseudo 1st order and pseudo 2nd order kinetic models equations respectively.

The adsorption pattern of CG acid onto NKA-II was studied by employing Freundlich and Langmuir isotherm on data obtained after attaining adsorption equilibrium at room temperature (Meng et al., 2023). The parameters and constants of both isotherm models can be calculated by using the following Eqs. (6), (7).

(6) $$\frac{C_{e}}{q_{e}}=\frac{1}{K_{l}q_{m}}+\frac{C_{e}}{q_{m}}$$

(7) $$\ln q_{e}=\frac{1}{n}\ln{\mathrm{qC}}_{e}+\ln K_{f}$$

The K_L_ and K_f_ are coefficient constants for Langmuir and Freundlich isotherm, n is Frrreundlich constant, whereas q_m_ represents maximum adsorption.

### 2.10 FESEM analysis with EDX

The surface morphology of dried WMR powder before any treatment and extracted residue of CG-water, CG-70 % Et, and CG-90 % Et powder was studied using FE-SEM (ZEISS-Sigma 360VP). The extract was plated on aluminum stubs and sputter-coated (EMS Quorum 150 TES, USA) with a 25 nm platinum layer before scanning. Multiple magnifications and imaging parameters were used, including the voltage of 5KV, 3.5 spot size, 30 μm objective aperture, and 8 mm working distance. The samples were coated with carbon adhesive tape (Al Hakkak et al., 2019).

### 2.11 FTIR analysis

FTIR analysis of purified samples (CG-water-p, CG-70 %-Et-p, and CG-90 %-Et-p) was conducted by using the FT-IR instrument (Carry-630 by Agilent Technologies). The samples were operated at 400–4000 cm^− 1^ wavelength with 2 cm^−1^ resolutions (Im et al., 2022) and spectra were arranged using origin-pro software (Náthia-Neves & Alonso, 2021a).

### 2.12 Thermal analysis

The thermal (TGA and dTGA) analysis of purified CG-water-p, CG-70 % Et-p, and CG-90 % Et-p was conducted using Perkin Elmer (TGA-7; a thermo-gravimetric analyzer). The 5 g of rotatory evaporator dried samples heated ranging from 30 to 600 °C in an inert atmosphere of nitrogen gas to avoid any oxidation (Usman et al., 2020). The heating rate and nitrogen gas flow rate for TGA analysis were maintained at 20 °C/ min and 20 mL/ min respectively (Lin et al., 2021). The weight loss of each sample was observed at initial degradation (T-onset) at 275 °C and further degradation (T-end set) was observed till 600 °C.

### 2.13 Statistical analysis

The results of each analysis are presented as the mean ± standard error of measurement. The data underwent statistical analysis using SPSS software (IBM, Armonk, NY, USA, version 21). ANOVA with the least significant difference (LSD) test was employed to assess the significant differences in the results. The statistical significance was measured by employing *p* < 0.01–0.05 level and results are labeled as ‘a’ representing significant *p* < 0.05 and ‘b’ representing highly significant p < 0.01, and “c” representing the super significant at *p* > 0.001.

## 3 Result and discussion

### 3.1 Extraction of chlorogenic acid

**Fig. 2:**
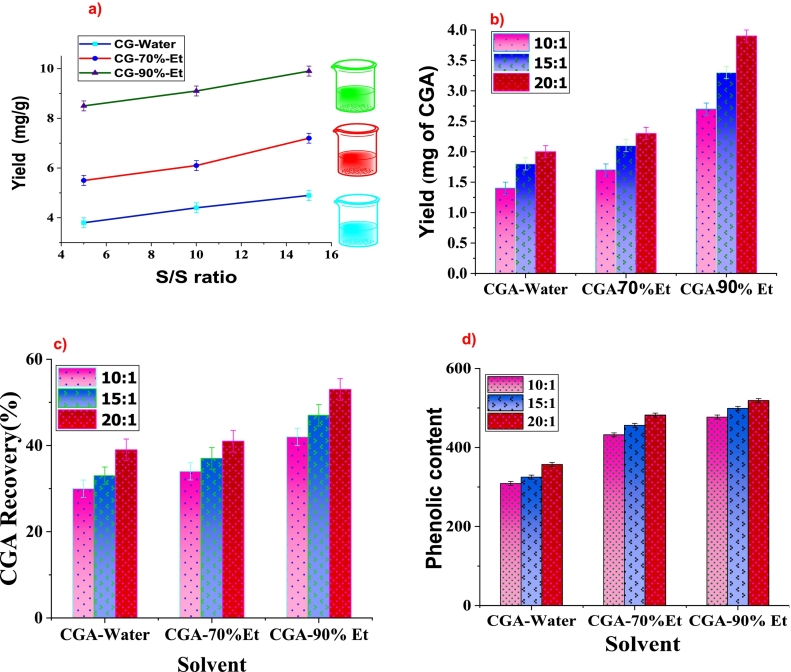
a) Impact of solvent nature on extraction efficiency, b) yield of CG acid, c) CG acid recovery, d) total phenolic content in watermelon rind.

The Chlorogenic (CG) acid was extracted using polar solvents with different degrees of polarity. It has good attraction with polar solvents and can be dissolved in it. The choice of a more polar solvent results in a high yield of extraction of CG acid. The extraction time is reduced using ultrasound waves that facilitate the mass transfer of CG acid into polar solvent. The yield of CG acid is strongly affected by the choice of solvent either single or mixture and the composition of solvents in the mixture (Náthia-Neves & Alonso, 2021a). Fig. 2a shows the effect of solvent choice on the yield of CG-acid extraction and the highest yield was observed in the case of using 70 % aqueous ethanol solution in the presence of ultrasound waves. Although water is a more polar solvent than ethanol, the literature revealed that the combination of water with other organic solvents like ethanol at 70 % composition would result in a higher extraction yield (Alves Filho et al., 2020)(Zeng et al., 2022).

It is noteworthy that the choice of the solvent mixture and its composition depends on the nature of the precursor because the effect of the solvent may vary depending on the type of substrate. Each substrate may have differences in CG-acid content and its bonding attraction with other phenolic content. Zeng et al. (2022) extracted higher CG acid content from tobacco leaves using 70 % aqueous ethanol solution while Náthia-Neves and Alonso, 2021a, Náthia-Neves and Alonso, 2021b got the highest CG acid content at 90 % aqueous ethanol solution (Zeng et al., 2022)(Náthia-Neves & Alonso, 2021a).

### 3.2 Extraction efficiency of chlorogenic acid

The CG acid extraction experiment was conducted at 10:1, 15:1, and 20:1 solvent-to-sample (S/S) ratio and the yield of CG acid extraction is shown in Fig. 2b. It is concluded that 90 % aqueous ethanol solution has an effectively high yield of CG acid extraction at 20:1 ratio. Table 1 shows the total phenolic content, yield, and recovery of CG acid extracted using water, 70 %, and 90 % ethanol solution. The highest purity and percent recovery of CG acid was found to be in the case of 90 % aqueous ethanol solution (Jiménez-moreno et al., 2019). At this composition, the solvent mixture helps in breaking the interactions of CG acid with other phenolic compounds and develops a strong attraction with CG acid content. This finding aligns with previous studies, particularly those by Jiménez-moreno et al. (**2019**) and Náthia-Neves and Alonso, 2021a, Náthia-Neves and Alonso, 2021b, which report that ethanol, particularly at high concentrations, is an effective solvent for extracting phenolic compounds due to its polarity and ability to break down cell walls for efficient compound release (Jiménez-moreno et al., 2019)(Náthia-Neves & Alonso, 2021a). A higher value than reported studies would be the reason for the cumulative effect of 90 % ethanolic extract and ultrasound waves that would make the release of CG acid content from watermelon rind.

**Table 1:** Total phenolic content, yield, and percent recovery of CG acid extracted using water, 50 %, and 70 % ethanol.

Samples	Solvent-to-Sample Ratio	Yield of CG acid (mg of CGA/g of raw material)		Percent Recovery		Total Phenolic Content (TPC) (mg GAE/g of extract)
			Av.		Av.	
Water	10/1	1.4 ± 0.5^a^	1.7	30^b^	34	309 ± 3.5^a^
	15/1	1.8 ± 0.5^a^		33^a^		325 ± 1.1^a^
	20/1	2.0 ± 0.5 ^a^		39^b^		357 ± 2.4 ^a^
70 % Et	10/1	1.7 ± 0.3^b^	2.0	34^a^	37	432 ± 5.3^a^
	15/1	2.1 ± 0.3^a^		37^a^		456 ± 3.3^b^
	20/1	2.3 ± 0.3^a^		41^b^		482 ± 5.5^a^
90 % Et	10/1	2.7 ± 0.2^b^	2.4	42^a^	48	477 ± 3.1^c^
	15/1	3.3 ± 0.2^a^		47 ^b^		499 ± 4.4^b^
	20/1	3.9 ± 0.2^c^		53^c^		519 ± 3.5^a^

(All data is significant and lies within the range of *p* < 0.05–0.001)

The percent recovery of CG acid was also observed higher using 90 % ethanol solution than water and 50 % ethanol, shown in Fig. 2c. The highest purity and percent recovery were observed with the 90 % aqueous ethanol solution, consistent with findings in the cited literature. Previous research has highlighted that ethanol at 90 % concentration enhances phenolic compound solubility and selectivity, leading to improved yield and recovery. Thus, the current results reinforce the effectiveness of 90 % ethanol as the optimal solvent for CG acid extraction, corroborating the conclusions of Jiménez-moreno et al. (2019) and Náthia-Neves and Alonso, 2021a, Náthia-Neves and Alonso, 2021b.

**Fig. 3:**
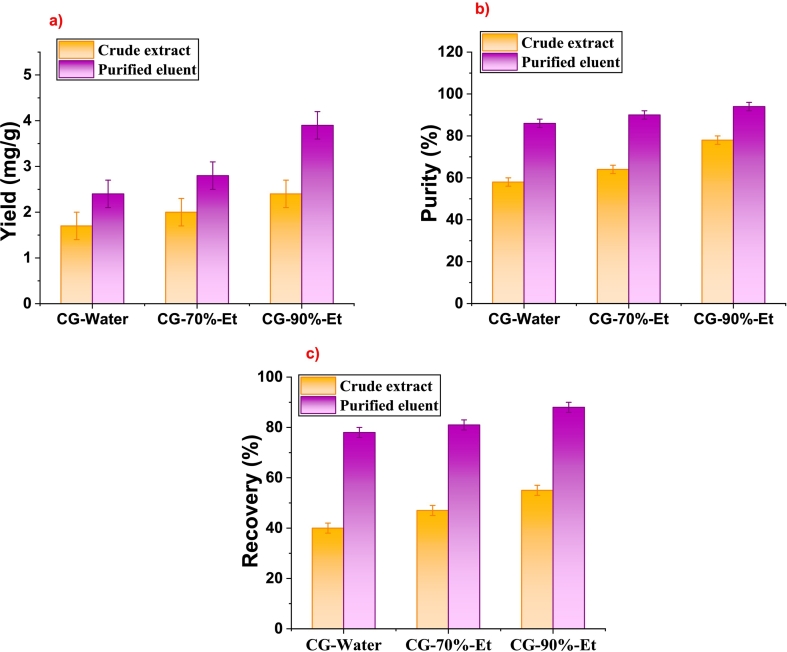
Purification of crude CG acid using MPAR (NKA-II), a) yield, b) purity (%), c) recovery (%) of crude extract and purified CG acid.

Purification of the crude CG acid extract using macroporous adsorption resin (MPAR, NKA-II) led to a notable enhancement in yield, purity, and recovery shown in Fig. 3(a, b, c). Specifically, the purification process doubled the CG acid yield, increasing from 2.4 mg/g to 4.6 mg/g dry weight when eluted with a 90 % ethanol solution. This significant improvement in yield and recovery can be attributed to the effective removal of other phenolic compounds and impurities present in the crude extract. The high adsorption efficiency of NKA-II resin for CG acid, as supported by literature, further validates this enhancement. Ultimately, the purification process resulted in a remarkable 94 % purity of CG acid.

A study utilizing NKA-II resin for chlorogenic acid (CGA) extraction reported an increase in CGA content to 22.17 %, with a recovery yield of 82.41 % (Wu et al., 2022). Similarly, another study on sunflower disks employed a combination of deep eutectic solvents and NKA-II resin, achieving a CGA extraction yield of 6.16 mg/g. Post-enrichment, the recovery efficiency reached 80.76 %, with a final purity of 75.84 % (Wu et al., 2022). Compared to these findings, the present study's purification using NKA-II resin resulted in a significantly higher CGA purity of 94 %, indicating the superior efficiency of the macroporous adsorption resin (MPAR) method in refining CGA from watermelon rind. However, variations in extraction sources, solvents, and process conditions should be considered when interpreting these differences. Table 2S shows the yield, purity, and recovery of CG acid after purification using NKA-II resin.

### 3.3 Total phenolic content (TPC)

As the watermelon rind also consisted of a series of phenolic compounds, its aqueous ethanolic extract, obtained after the extraction procedure, was subjected to total phenolic content (TPC) determination. The result of the total phenolic content of watermelon rind is shown in Fig. 2d. The resulting profile of the TPC of watermelon rind showed that its value ranges from 379 to 519 mg/g of CG extract using different extracting solvents. The highest TPC was found to be extracted with 90 % ethanolic solution at a 20:1 solvent-to-sample ratio. Besides the 20:1 ratio of each solvent extracted a higher TPC value than other solvent-to-sample ratios under the same extraction condition. This is attributed to higher solvent concentrations can easily interact with biomass during sonoporation and reduce the risk of increasing viscosity of solution and easy breakage of ester linkage (Jiménez-moreno et al., 2019). The TPC of watermelon rind extracted from this study is significantly higher than the TPC found in pineapple but lower than apple 424 mg GAE/g and 2721 mg GAE/g (Dai et al., 2018).

### 3.4 Adsorption isotherm and kinetics

**Fig. 4:**
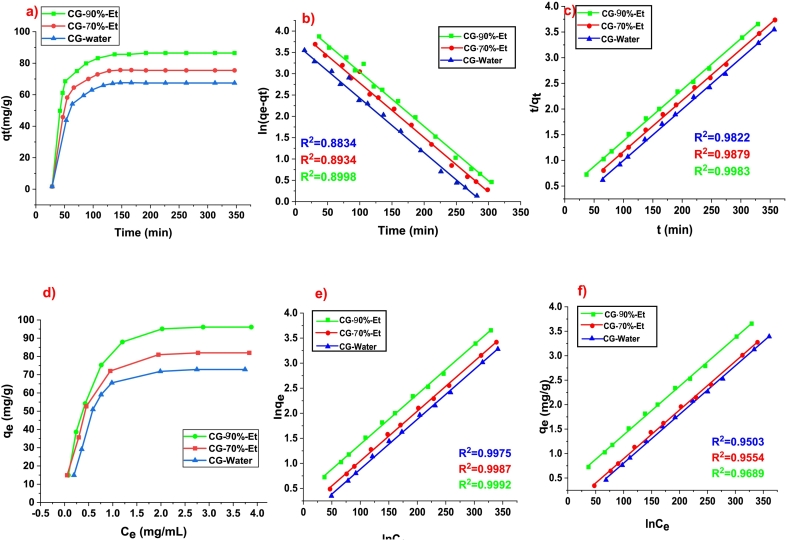
a) Adsorption rate of extracted chlorogenic acid, b) kinetics of psuedo 1st order, c) 2nd order, d) adsorption isotherm, fitting of adsorption isotherm; e) langmuir and f) freundlich.

The kinetics of adsorption of CG-water, CG-70 %-Et, and CG-90 %-Et were studied and demonstrated in Fig. 4a. All samples have an abrupt increase in adsorption rate up to 1 h followed by a slow increase and then attained a plateau position after passing 2 h. The CG-90 %-Et has a comparatively greater rate of adsorption rate and adsorbed about 80 % of the concentration within the first 2 h than CG-water (40 %) and CG-70 %-Et (60 %). The kinetic of adsorption was studied by applying pseudo 1st and 2nd order and its fitting is shown in Fig. 4 (b and c). The concerning constant of each model was calculated using the aforementioned equations and given in Table 3S. The regression coefficient constant (R^2^) has more closer value to 1 in the case of pseudo 2nd order (0.9822–0.9983) than pseudo 1st order (0.8834–0.8998). So pseudo 2nd order model is applicable to study the kinetics of adsorption (Hou & Zhang, 2021).

The adsorption isotherm plotted between “q_e_” verses “C_e_” to investigate the distribution of CG acid between two phases (CG extract and solid slurry of NKA-II) when adsorption capacity reaches equilibrium. The adsorption isotherm of each crude extract (water, 70 %-Et, and 90 % -Et) of CG acid sample on NKA-II is given in Fig. 4d, and it shows the relationship of concentration of adsorbed CG acid (C_e_) with adsorption capacity at the equilibrium point (q_e_). As the concentration of adsorbed CG acid increased, the adsorption capacity also increased gradually at the start and attained a saturated situation of adsorption when the conc. of CG acid adsorbed maximum (Meng et al., 2023). The CG-70 %-Et showed higher adsorption capacity than CG-water and CG-70 %-Et. The fitting of adsorption isotherm was calculated by employing Langmuir and Freundlich equations and shown in Fig. 4 (e and f). The parameters and respective constants of both equations were calculated and described in Table 4S. The comparative analysis showed that the value of R^2^ for the Langmuir equation is nearer to 1 than the Freundlich equation indicating the more fitting of uni-layer adsorption theory by Langmuir (Meng et al., 2023)(Fu et al., 2021).

The adsorption efficiency of chlorogenic acid (CG) extracted using different solvents; water, 50 % ethanol, and 70 % ethanol was evaluated based on equilibrium adsorption capacity (qe) and adsorption kinetics. From the equilibrium isotherm data (Figure a), the highest adsorption capacity was observed for CG extracted with 70 % ethanol (CG-70 %-Et), reaching approximately **80–90 mg/g** at higher equilibrium concentrations (Ce). This was followed by CG-50 %-Et, which exhibited a moderate adsorption capacity of **60–70 mg/g**, while CG-Water showed the lowest adsorption efficiency, ranging from **40 to 50 mg/g**. The kinetic analysis (Figure d) revealed that CG-70 %-Et reached equilibrium faster (∼200 min) with the highest adsorption rate, whereas CG-50 %-Et required approximately 250 min, and CG-Water took nearly 300 min to stabilize with the lowest adsorption. The adsorption behavior followed a pseudo-second-order kinetic model, suggesting chemisorption as the dominant mechanism.

These findings align with and extend the results of Hou and Zhang (2021), who reported that the adsorption capacity of CG acid on macroporous resins varied between **65 and 85 mg/g**, depending on solvent polarity and resin type (Hou & Zhang, 2021). The higher adsorption observed in our study for CG-70 %-Et is consistent with their findings, which emphasized that ethanol-water mixtures enhance solute diffusion and interaction with the resin, leading to improved adsorption. Similarly, Meng et al. (2023) investigated the adsorption of CG acid and reported equilibrium adsorption values between **50 and 75 mg/g**, with optimal adsorption occurring in ethanol-water mixtures. However, our study demonstrated a slightly higher adsorption capacity (**80–90 mg/g**) for CG-70 %-Et, indicating that the selection of ethanol concentration and resin type plays a crucial role in maximizing adsorption efficiency (Meng et al., 2023). Compared to both studies, our results confirm that using 70 % ethanol significantly enhances adsorption efficiency by improving solvent polarity balance, facilitating better CG acid extraction, and enhancing interaction with the macroporous resin.

### 3.5 UV spectro-photometry analysis

**Fig. 5:**
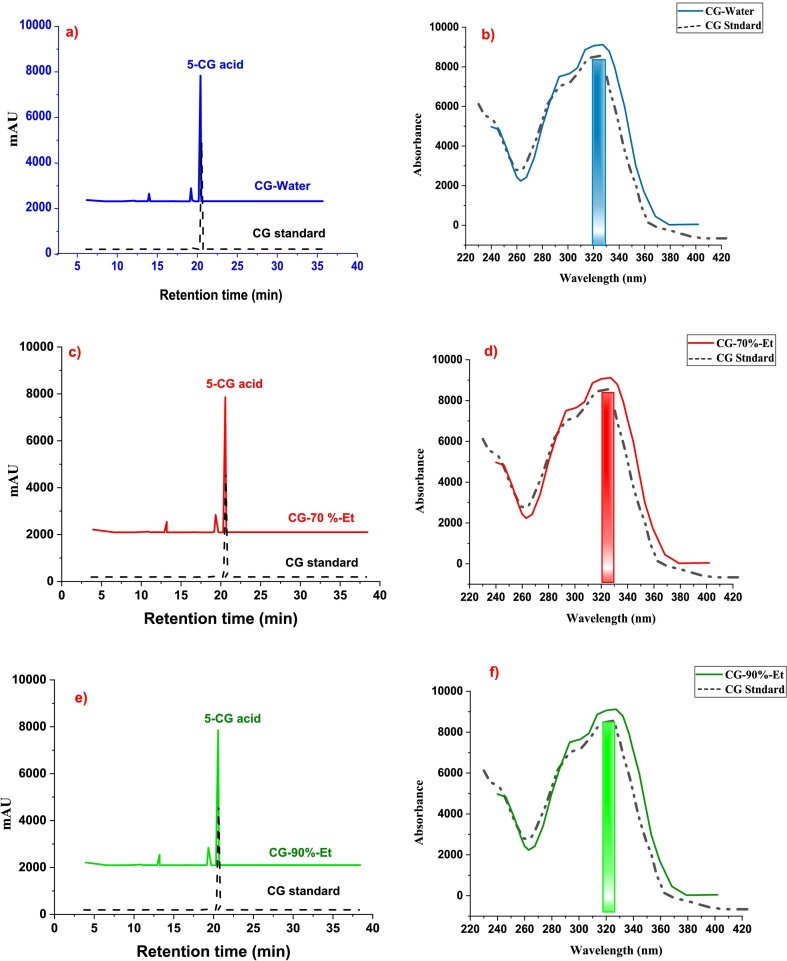
(a, c, and e) HPLC chromatogram of CG acid eluted with water, 70 % Et and 90 %-Et solvents, (b, d, and f) UV absorbance of CG acid extracted using water, 70 % Et, and 90 % Et solutions.

Each CG acid extract sample with a 20:1 solvent-to-sample ratio, which produced a high yield of CG acid, was also subjected to UV spectrophotometric analysis to get its absorbance peak in the spectrum (Zeng et al., 2022)(X. Wang et al., 2019). Fig. 5 (b, d, and f) shows the spectra of UV absorbance of CG acid extracted using water, 70 %-Et, and 90 %-Et solutions respectively. All three samples show absorbance in the same region of wavelength with a little difference ranging from 325 to 329 nm wavelength. The CG acid gives a broad band in this region of wavelength. The results are in line with the literature and confirm the CG acid extraction (Rivera-Mondragón et al., 2019)(Zeng et al., 2022).

### 3.6 HPLC analysis

In the HPLC, the identification and isolation of components from a sample mixture are based on the elution solvent and retention time. The retention time for the separation of a specific component depends on the choice of solvent. A polar solvent can elute the phenolic component more quickly due to its strong bonding attractions with the desired component. The HPLC chromatogram of the crude extract of CG acid, Fig. 1S, contains more peaks for undesired phenolic components in addition to the peak for CG acid, while that of the purified sample is shown in Fig. 5 and has a coincidence with the standard 5-CG acid chromatogram, (Zeng et al., 2022)(X. Wang et al., 2019). Fig. 5 (a, c, and e) shows the HPLC chromatogram of CG acid eluted with water, 70 % Et, and 90 %Et solvent respectively.

All chromatograms showed the presence of its most abundant isomer (5-CG acid) with the highest intensity among other peaks, these two peaks correspond to the presence of other CG acid isomers. These isomers would be the monomeric (3-CG and 4-CG) form of CG acid because the dimeric (3,5-di-O-caffeoylquinic acid and 4,5-di-O-caffeoylquinic acid) form of CG acid compounds eluted at longer retention time than monomeric isomers. Besides, the 5-CG acid took longer 20.9 min to elute with water than 70 %-Et and 90 %-Et solvents (Hou & Zhang, 2021)(Meng et al., 2023). Several reported studies revealed that aqueous ethanolic solvents (70 % and 90 %) are more promising solvents in CG acid extraction than pure water (Náthia-Neves & Alonso, 2021a)(Zeng et al., 2022).

**Fig. 6:**
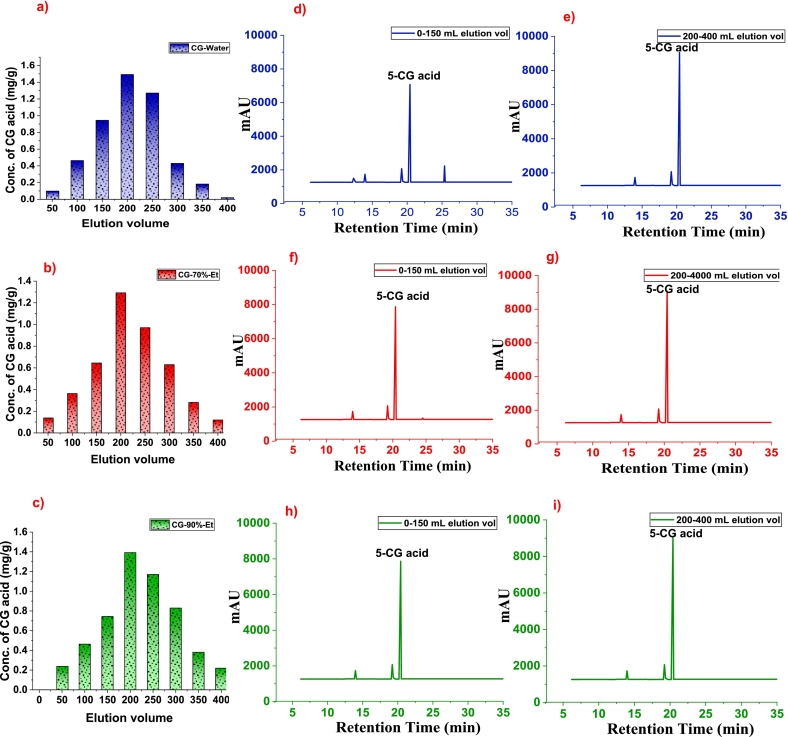
(a, b, and c) conc. of CG acid after elution volume (0–400 mL vol), (d & e), (f & g), and (h & i) HPLC profile for 0–150 mL and 200–400 mL volume of eluent for CG-water, CG-70 %-Et and CG-90 %-Et.

During purification, the adsorbed CG acid of each sample was desorbed using their respective solvents, and multiple fractions after each 50 mL volume of desorbed eluent (DSE) were obtained in each case. The conc. of CG acid determined in each eluent fraction is shown in Fig. 6 (a, b, and c). The HPLC profile of purified CG acid for 0–150 mL and 200–400 mL volume of eluent for CG-Water, CG-70 %-Et, and CG-90 % Et is shown in Fig. 6 (d & e), (f & g), and (h & i) respectively.

It is noteworthy in Fig. 6 (d & f) that a peak for a small fraction of undesired compounds was also eluted in addition to 5-CG acid in 0–150 mL volume of DSE. The undesired compound would be any water-soluble sugars or less polar phenolic components that were being eluted. Additionally, two peaks at a shorter retention time corresponding to its isomeric forms were eluted in all elution fractions. Contrarily, the later fractions of DSE (200–400 mL volume) eluted only 5-CG acid with higher intensity peaks rather than in 0–150 mL volume of DSE. The elution fraction with higher volume didn't contain any undesired component because polar compounds usually don't elute in later fractions and thus we get pure fractions. The 90 %-Et solvent played a significant role in eluting the pure CG acid in all fractions (0–400 mL vol.) with varied peak intensities without any undesired component peak, indicating the efficient elution in comparison to water and 70 %-Et solvents.

The extraction and purification of CG acid are driven by solvent polarity and chromatographic behavior. Aqueous ethanol (70 % and 90 %) outperformed water due to its balanced polarity, enhancing CG acid solubility and minimizing interference. The longer retention time of 5-CG acid in water (20.9 min) indicates weaker interactions with the stationary phase, slowing elution. During purification, NKA-II resin selectively adsorbed CG acid, while 90 % ethanol efficiently eluted pure fractions across all volumes (0–400 mL), ensuring minimal impurities. These results confirm ethanol's superiority in phenolic compound extraction and purification.

### 3.7 Liquid chromatography-electrospray ionization/mass spectrometry (LC-ESI/MS) analysis of CG acid

**Fig. 7:**
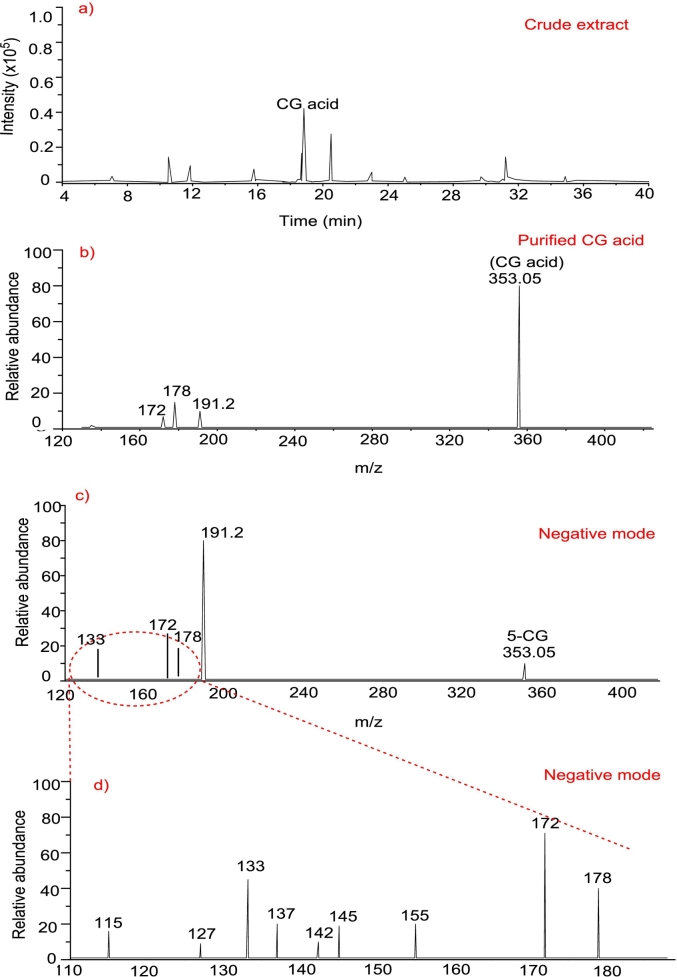
a) LC-ESI/MS spectra of crude extract of CG acid, b) LC-ESI/MS spectra of purified CG acid using 90 %-Et, c) LC-MS/MS fragmentation pattern of 5-CG acid in the negative mode of ions mobility, d) Zoom in spectra of LC-MS/MS fragmentation pattern of m/z 191 ion ranging from 115 to 180.

The LC-ESI/MS spectra of crude extract of CG acid, shown in Fig. 7 (a) also contain the peaks corresponding to the presence of other phenolic compounds present in watermelon rind extract. All phenolic compounds have different absorbances depending on their concentration present in the extract. Contrarily, the LC-ESI/MS spectrum of purified CG acid, shown in Fig. 7 (b), contains only one peak for the most prevalent isomer (5-CG acid) revealing the presence of singly charged molecules in negative ion modes ([M-H]-). The calculated *m*/*z* values for the charged ions resulting from deprotonated species of CG acid are 353.0878, and the findings indicated that the peak corresponding to this ion was observed at 353.08.Chlorogenic acid (5-CG), being the predominant mono-caffeoylquinic acid in nature, was selected for studying the fragmentation pattern in this research. The dissociation behavior of 5-CG acid isomers was investigated using negative ionization is shown in Fig. 7 (c).

Negative ionization is generally preferred for phenolics due to its higher sensitivity compared to positive ionization, making it a common choice for CG acid MS/MS analysis. In the negative ion mode, three principal product ions were detected at m/z values of 191, 178.8, and 172.6. The ion at m/z 191 was identified as quinic acid, resulting from the cleavage of the ester bond linking quinic and caffeic acid components. This ion emerged as a significant peak across all three isomers, reflecting the ester bond's susceptibility to cleavage during LC-MS/MS processes, possibly facilitated by the carboxylic acid group's readiness for ionization in the negative ion mode (Salman et al., 2018).

The quinic acid product ion (m/z 191) is further dissociated in Fig. 7 (d). The formation of a product ion at m/z 172.6 through the loss of a water molecule was anticipated, given the quinic acid moiety's multiple hydroxyl groups, which offer numerous sites for water loss. This finding is consistent with prior reports in the literature for 5-CG acids and other chlorogenic acid isomers. Additionally, product ions at m/z 155.0 and 137.0 resulted from further water molecule loss from the m/z 172.6 ion, while the ion at m/z 115 was produced by the elimination of carbon dioxide. The ion at m/z 178.8 was traced back to the caffeic acid segment of the molecule, a fragmentation pattern was also reported for 5-CQA and observed in other chlorogenic acids where the cinnamic acid moiety is released during MS/MS analysis in negative ion mode. This ion further loses a carbon dioxide unit to form the product ion seen at m/z 133, aligning with documented fragmentation behaviors in the literature (Ayouaz et al., 2021).

### 3.8 FESEM analysis with EDX

**Fig. 8:**
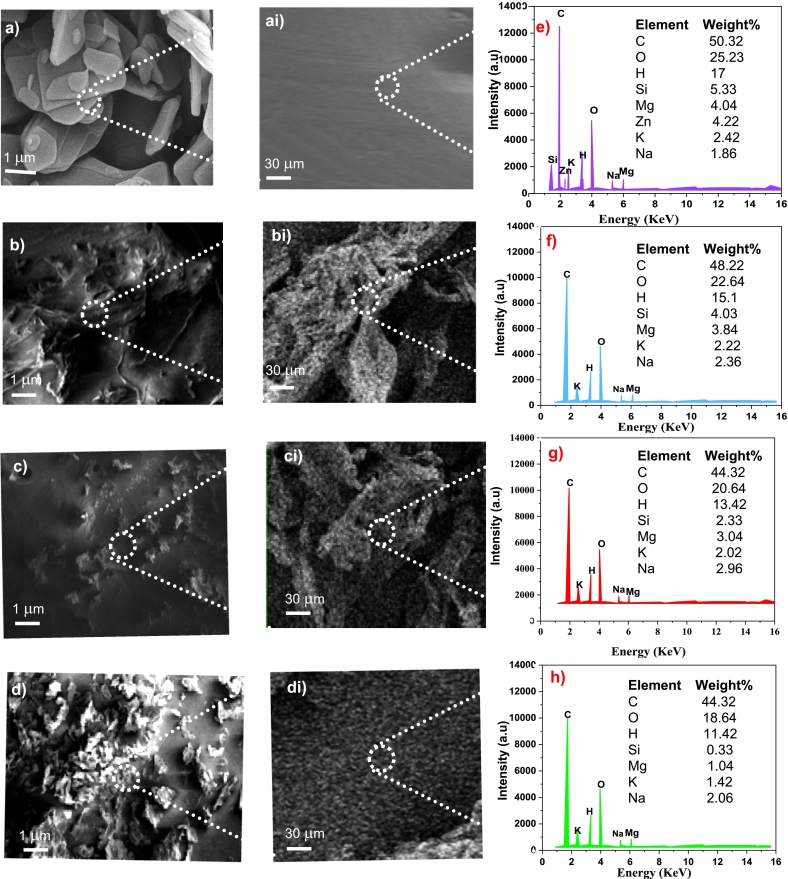
(a and ai) FESEM analysis of the watermelon rind (WMR) powder before extractions, (b and bi), (c and ci), and (d and di) FESEM analysis of residue of WMR extracted using USAE with water, 70 %-Et and 90 %-Et showing porous structures more profoundly and precisely (e, f, g, and h) EDX of WMR powder and the extracted residue treated with water, 70 %-Et and 90 %-Et.

The FESEM analysis of the WMR powder before extractions is shown in Fig. 8 (a and ai). It is noteworthy to know that the surface of WMR powder has a small discrete pellet-like texture and its surface is smooth showing the compact bonding of structural components. The USAE treatment of WMR powder using water, 70 % Et, and 90 % Et is shown in Fig. 8 (b and bi), (c and ci), and (d and di) respectively. The USAE treatment of WMR has resulted in the roughening of surface extracted residue that shows that the hydrogen bonding of phenolic and structural components breaks down and releases its mass content into the solution making it more viscose. Fig. 8 (bi, ci, and di) shows the surfaces of extracted residue showing porous structures more profoundly and precisely (Náthia-Neves & Alonso, 2021a). The impact of the selection of solvent for USAE is evident from the highly porous surface observed in extracting CG acid with 90 % Et than water and 70 % Et. The more polar nature of 90 % Et solvent has more tendency to interact more strongly with CG acid and the assistance of ultrasound waves facilitated the deep penetration and extraction of CG acid. The results coincide with the literature and are justified (Akpabli-Tsigbe et al., 2022; S. Chen et al., 2024; Náthia-Neves & Alonso, 2021b; Wan et al., 2024).

The elemental analysis of WMR powder and the extracted residue treated with water, 70 % Et and 90 % Et are shown in Fig. 8 (e, f, g, and h) respectively. The compositional profile shows that WMR powder is enriched with C, O, and H percentages along with the presence of minerals and silicon impurities are minors. The ultrasonic treatment of WMR powder resulted in a significant reduction in the percentage of silicon along with C, H, and O percentages. This reduction is more pronounced in treatment with 90 % Et than in water and 70 % Et. It is evident from the literature that 70 % ethanol solution has a good dissolution strength for impurities in addition to the phenolic compounds.

### 3.9 FTIR analysis

**Fig. 9:**
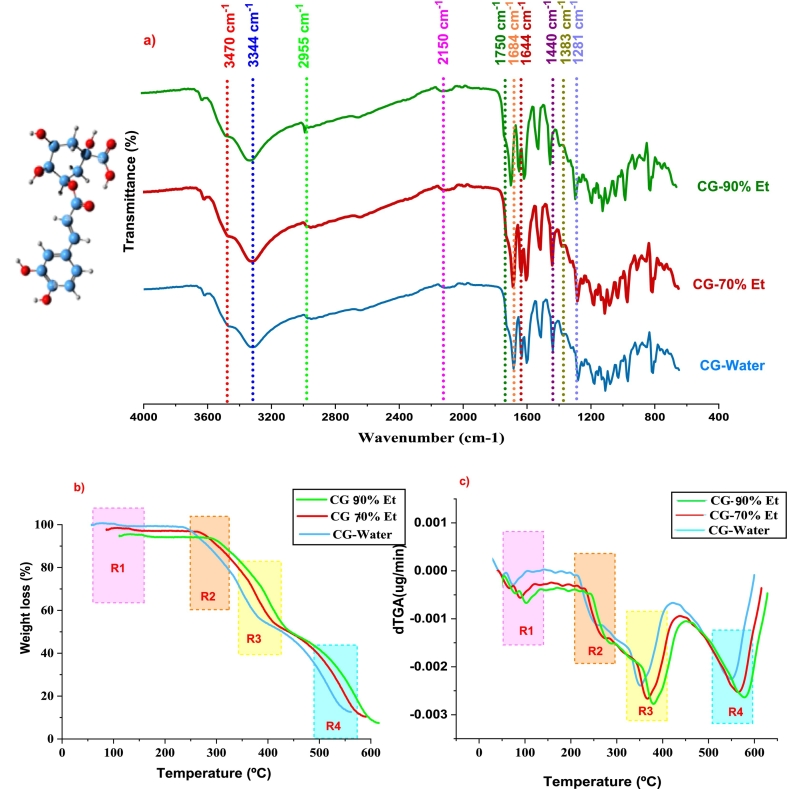
a) FTIR spectra of purified CG-acid using Water, CG-70 % Et, and CG-90 % Et, b) TGA, c) dTGA of purified CG-acid using Water, CG-70 % Et, and CG-90 % Et.

The FTIR spectra of transmittance bands of all functional groups of CG-acid extracted with different solvent compositions are shown in Fig. 9a. All characteristic peaks for each functional group, shown in Table 5S, fall within the range of wavelength and exhibit similar spectra with minor differences in absorption bands. It is evident from the spectra that no significant difference lies in vibration bands of functional groups of CG acid due to the influence of extracting solvent's composition (Zohreh, 2020). The CG acid extraction was confirmed by the presence of a characteristic peak that appeared at 3470–3474 cm^−1^ for stretching vibrations of hydrogen bonding between terminal OH groups.

Moreover, the appearance of absorption peaks for O—H and C—H stretching vibrations at 3344–3352 cm^−1^and 2955–2956 cm^−1^ respectively support the efficient extraction of CG acid (Alves Filho et al., 2020). The presence of C=C bond conjugation in CG acid is confirmed by the appearance of the peak for bending and stretching vibrations at 2150–2152 cm^−1^ and 16,643–1644 cm^−1^ respectively. The peaks for stretching vibrations of another significant functional group C=O of carboxylic acid and C=O of ester linkage appeared at 1748–1750 and 1683–1787 cm^−1^ respectively while the stretching vibrations of C-O-C appeared at 1280–1283 cm^−1^. The stretching vibrations of the basic skeletal peak of the C—C aromatic ring and bending vibrations of C—H appeared at 1440 and 1383–1387 cm^−1^ respectively (Zeng et al., 2022).

### 3.10 Thermal analysis

The thermal characteristics of purified CG-Water, CG-70 % Et, and CG-90 % Et can be elucidated based on bond dissociation at specific temperature intervals. The Thermogravimetric Analysis (TGA) and derivative Thermogravimetric Analysis (dTGA) curves of chlorogenic acid reveal four primary zones: dehydration, ester cleavage, decomposition, and combustion (Rizwan et al., 2021). In the dehydration zone (50–150 °C), a 5–10 % weight loss occurs due to the elimination of water content within the samples. The ester cleavage zone (200–275 °C) exhibits a more substantial weight loss (approximately 20–25 %) due to the cleavage of ester linkages between caffeic acid and quinic acid. This zone experiences a notable transformation involving the removal of the carbonyl group from the ester linkage, generating phenol-derivative subunits with varying molar weights.

In the decomposition zone (325–425 °C), a significant weight loss of 32–39 % is observed as each phenolic subunit undergoes degradation, resulting in a loss of thermal stability (Liu et al., 2023). This zone is marked by structural alterations involving decarboxylation, leading to the removal of carbon dioxide. The TGA and dTGA graph of purified samples (CG-Water, CG-70 % Et, and CG-90 % Et) are shown in Fig. 9 (b and c) During the combustion zone, heating the subunits to 600 °C results in their conversion into elemental forms, leaving behind an ash residue. The peak degradation temperature indicates that CG-90 % Et initiates degradation at 375 °C and exhibits greater stability compared to CG-70 %Et and CG-Water. Contrarily, CG-90 % Et demonstrates relatively lower weight loss across all zones, indicative of enhanced stability in comparison to the other counterparts. The weight loss of each sample at a specific temperature zone is enlisted in Table 6S.

### 3.11 Limitations and future work

While the study highlights the effectiveness of ultrasound-assisted extraction (USAE) and macroporous adsorption resin (MPAR) purification for recovering chlorogenic acid from watermelon rind, limitations include the restricted selection of solvents, challenges in scalability, high energy consumption, and limited stability analysis, and lacks industrial-scale validation. Future research should explore greener extraction methods, nanoencapsulation for stability, biorefinery approaches for multi-compound recovery, and AI-driven process optimization. Further investigations into bioavailability, therapeutic potential, and large-scale economic feasibility are essential to enhance commercial applicability.

## 4 Conclusion

This study investigates the sustainable recovery of bioactive components from agro-food waste by extracting and purifying chlorogenic (CG) acid from watermelon rind (WMR). The extraction was conducted using ultrasound-assisted extraction (USAE) at 40 kHz and 200 W for 30 min, followed by purification with macroporous adsorption resin (MPAR) under optimized conditions. Three solvents water, 70 %, and 90 % ethanol were used to evaluate their impact on extraction efficiency, purity, and recovery. The extracted CG acid was analyzed through UV spectrometry, HPLC, and FESEM, while the purified product was characterized using FTIR and thermal analysis. FESEM revealed a porous structure in the dried CG acid residue, confirming successful extraction. The MPAR purification significantly enhanced the yield to 4.6 mg/g dry weight, with purity levels of 48–59 mg CGA/g when using 90 % ethanol. Recovery improved to 88 %, with an initial purity range of 70–94 %. The choice of solvent, along with the selection of a suitable resin type, played a crucial role in optimizing extraction efficiency, yield, and recovery of CG acid from WMR.

## CRediT authorship contribution statement

**Sobia Naseem:** Writing – original draft, Visualization, Software, Methodology, Investigation, Formal analysis, Data curation. **Muhammad Rizwan:** Writing – review & editing, Supervision, Formal analysis, Data curation, Conceptualization. **Wahidah H. Al-Qahtani:** Supervision, Funding acquisition, Conceptualization. **Ayesha Sadiqa:** Conceptualization. **Awais Ahmad:** Supervision, Conceptualization.

## Declaration of competing interest

The authors declare that they have no known competing financial interests or personal relationships that could have appeared to influence the work reported in this paper.

## Data Availability

The authors are unable or have chosen not to specify which data has been used.

## Appendix A Supplementary data

Supplementary material Image 1
