# Supplementary material for: Purification of ultrasonic assisted extracted chlorogenic acid from Citrullus lanatus rind using macroporous adsorption resin (MPAR)
Source: Food Chem X. 2025 Mar 19;27:102374. doi: 10.1016/j.fochx.2025.102374 (PMC11984621; doi:10.1016/j.fochx.2025.102374)
Supplement: Supplementary file 1 — Supplementary material [file mmc1.docx]

**Purification of Ultrasonic Assisted Extracted Chlorogenic Acid from Watermelon (*Citrullus lanatus*) Rind using Macroporous Adsorption Resin (MPAR)**

**Table 1S: Physical properties of NKA-II**

| **Properties** | **NKA-II** |
| --- | --- |
| Nature | Polar |
| Water content (%) | 55 |
| Average aperture (nm) | 14-16 |
| Specific surface area (m^2^/g) | 150-190 |
| Saturated adsorption capacity(mg/g) | 7.5 |
| Desorption rate (%) | 70.2 |
| Average Pore Size (nm) | 20–22 nm |

**Table 2S: Yield, purity, and recovery of CG acid after purification using NKA-II resin**

| **Samples** | **Yield of CG acid** | | **Purity (%)** | | **Recovery of CG acid (%)** | |
| --- | --- | --- | --- | --- | --- | --- |
|  | mg of CG acid/g of raw material | |  |  |  |  |
|  | Crude  Extract | Purified  Eluent | Crude  extract | Purified  eluent | Crude extract | Purified  Eluent |
| Water | 1.7^a^ | 2.4^a^ | 58^b^ | 84 | 40^a^ | 68^b^ |
| 70%-Et | 2.0^b^ | 2.8^a^ | 64^b^ | 90^b^ | 47^b^ | 76^a^ |
| 90%-Et | 2.4^c^ | 4.6 | 70^a^ | 94^b^ | 55^a^ | 88^b^ |

(All data is significant and lies within the range of p<0.05-0.001)

**Table 3S: The parameters of adsorption kinetics of CG acid on NK-IIA resin**

|  | **Pseudo 1^st^ order** | | |  | **Pseudo 2^nd^ order** | | |  |
| --- | --- | --- | --- | --- | --- | --- | --- | --- |
|  | **Qe** | **K_1_** | **R_1_^2^** | **Equation** | **Qe** | **K _2_** | **R_2_ ^2^** | **Equation** |
|  | mg/g | 1/min |  | $\ln\left( q_{e}-q_{t} \right)=$ | mg/g | g/mg/min |  | $\frac{t}{q_{t}}=$ |
| **CG-Water** | 22.19 | 1.456 | 0.8834 | -0.0145*t* +  4.4903 | 41.30 | 3.029 | 0.9822 | 0.0203*t* + 0.3324 |
| **CG-70 %-Et** | 28.23 | 1.986 | 0.8934 | -0.0198*t* +  5.6732 | 53.46 | 3.634 | 0.9879 | 0.0502*t* + 0.4356 |
| **CG-90 %-Et** | 36.40 | 2.098 | 0.8998 | -0.0148*t* +  6.4466 | 61.76 | 4.594 | 0.9983 | 0.0658*t* + 0.4976 |

Table 4S: The parameters of adsorption isotherms of CG acid on NKA-II resin

| **Samples** | **Langmuir** | | |  | **Freundlich** | | |  |
| --- | --- | --- | --- | --- | --- | --- | --- | --- |
|  | **Q_o_** | **K_L_** | **R^2^** | **Equation** | **Kf** | **1/n** | **R^2^** | **Equation** |
|  | mg/g | L/mg |  | $\frac{C_{e}}{Q_{e}}=$ | mg/g.(L/mg)^1/n^ |  |  | $lnq_{e}=$ |
| **CG-Water** | 14.23 | 6.94 | 0.9975 | 0.0089C_e_+  1.0642 | 24.0 | 0.6776 | 0.9505 | 0.2467ln*C*e +  2.6767 |
| **CG-50 %-Et** | 15.33 | 6.83 | 0.9987 | 0.0092C_e_+  1.04563 | 26.8 | 0.7185 | 0.9554 | 0.2343ln*C*e +  3.5326 |
| **CG-70 %-Et** | 16.46 | 6.34 | 0.9992 | 0.0096C_e_+  1.0659 | 27.1 | 0.7636 | 0.9689 | 0.2589ln*C*e +  4.2376 |

**Table 5S: FTIR spectra of purified CG acid extracted with water, 50 %-Et, and 70 %-Et solvents**

| **Absorbance** | | | **Functional groups** |  |  |
| --- | --- | --- | --- | --- | --- |
| **CG-Water** | **CG-50 %-Et** | **CG-70 %-Et** |  |  |  |
| 3470 | 3472 | 3474 | **H-bonding between terminal OH** |  |  |
| 3344 | 3348 | 3352 | **O-H stretching vibrations** |  |  |
| 2955 | 2956 | 2955 | **C-H stretching vibrations** |  |  |
| 2150 | 2150 | 2152 | **C=C bond Conjugation bending vibration** |  |  |
| 1750 | 1750 | 1748 | **C=O Stretching of carboxylic acid of CGA** |  |  |
| 1684 | 1683 | 1687 | **C=O vibrations of ester of CGA** |  |  |
| 1644 | 1643 | 1644 | **C=C bond Conjugation stretching vibration** |  |  |
| 1440 | 1440 | 1440 | **C-C Aromatic stretching** |  |  |
| 1383 | 1385 | 1387 | **C-H bending Vibrations** |  |  |
| 1281 | 1280 | 1283 | **C-O-C carboxylic stretching vibrations** |  |  |

**Table 6S: TGA and dTGA of purified CG acid extracted with water, 50 %-Et, and 70 %-Et solvents**

| **Sample** | **Weight loss (%)** | | | | **Peak**  **Degradation (ºC)** | **Residue** |
| --- | --- | --- | --- | --- | --- | --- |
|  | **Dehydration zone** | **Ester cleavage zone** | **Decomposition zone** | **Combustion zone** |  |  |
|  | **50-150 ºC** | **200-275 ºC** | **325-425 ºC** | **475-600 ºC** |  |  |
| **CG-Water** | 10 | 25 | 39 | 17.2 | 350 | 91 |
| **CG-50 % Et** | 7 | 23 | 36.4 | 15.4 | 363 | 81 |
| **CG-70 % Et** | 5 | 20 | 32.6 | 12.6 | 375 | 69 |

**Figure 1S: HPLC chromatogram of crude extract of CG acid**
